# miRNA‐194 predicts favorable prognosis in gastric cancer and inhibits gastric cancer cell growth by targeting CCND1

**DOI:** 10.1002/2211-5463.13125

**Published:** 2021-03-23

**Authors:** Jingjie Wang, Meixin Zhang, Xinhui Hu, Jiajun She, Ruonan Sun, Shanshan Qin, Dandan Li

**Affiliations:** ^1^ Hubei Key Laboratory of Embryonic Stem Cell Research School of Basic Medical Sciences Hubei University of Medicine Shiyan China; ^2^ Laboratory of Tumor Biology School of Biomedical Engineering Hubei University of Medicine Shiyan China

**Keywords:** CCND1, cell cycle, gastric cancer, miR‐194, prognostic biomarker

## Abstract

MicroRNAs (MiRNAs) play critical roles in regulating target gene expression and multiple cellular processes in human cancer malignant progression. However, the function of miR‐194 in gastric cancer (GC) remains unclear and controversial. In this study, we identified a series of miRNAs that can serve as prognostic biomarkers for GC by analysis of miRNA expression using The Cancer Genome Atlas data. Among them, miR‐100, miR‐125b, miR‐199a, and miR‐194 were the four most promising prognostic biomarkers in GC due to their significant associations with various clinical characteristics of patients. miR‐100, miR‐125b, and miR‐199a predicted poor prognosis in GC, while miR‐194 predicted favorable prognosis in GC. We also provide the first comprehensive transcriptome analysis of miR‐194 in GC. Our data suggest that miR‐194 tends to regulate target genes by binding to their 3′ UTRs in a 7‐mer‐A1, 7‐mer‐m8, or 8‐mer manner. KEGG pathway analysis showed that the cell cycle was one of the pathways most affected by miR‐194 in GC. Moreover, CCND1 was shown to be a novel target gene of miR‐194 in GC. Additionally, downregulation of CCND1 by miR‐194 in GC further led to cell growth inhibition and cell cycle arrest. In conclusion, miR‐100, miR‐125b, miR‐199a, and miR‐194 may have potential as prognostic and diagnostic biomarkers for GC. miR‐194 suppresses GC cell growth mainly through targeting CCND1 and induction of cell cycle arrest.

## Graphical abstract

Through global microRNAs expression analysis, we identified miR‐100, miR‐125b, miR‐199a, and miR‐194 as the 4 most promising biomarkers in gastric cancer (GC). miR‐100, miR‐125b, and miR‐199a predicted poor prognosis, while miR‐194 predicted favorable prognosis in GC. RNA‐seq analysis revealed CCND1 as a potential target gene of miR‐194‐5p in GC. miR‐194‐5p may suppress GC cell growth through targeting CCND1 and induction of cell cycle arrest.

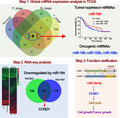

**Abbreviations**
GC: gastric Cancer
GEO: gene expression omnibus
MiRNA: microRNA
qRT–PCR: quantitative real‐time PCR
TCGA: the cancer genome atlas

Gastric cancer (GC) is the fourth most diagnosed type of cancer and the third most common cause of cancer‐related death worldwide [1]. Approximately 989 600 people worldwide are diagnosed with stomach cancer each year. More than half of those cases are diagnosed in East Asia [2]. However, many patients with GC are diagnosed with advanced malignant proliferation, extensive invasion, and distant metastasis. Hence, the 5‐year survival rate of patients with advanced GC was still unsatisfactory. Characteristic progressive tumorigenesis and distant metastasis may contribute to the overall poor prognosis a lot in GC [3]. Therefore, it is essential to develop accurate biomarkers to predict cancer stage or reflect an individual's cancer risk, which would be very useful for reducing GC mortality.

Mature microRNAs (miRNA) are a group of small noncoding RNAs with 19–24 nucleotides. Mature miRNAs bind to the specific regions in the 3′‐UTRs of the target mRNAs and negatively regulate their expression at post‐transcription level [4]. Increasing studies have shown that miRNA plays a very important role in tumorigenesis by affecting multiple cellular processes, such as epithelial–mesenchymal transition, cell invasion, cell apoptosis, and cell proliferation [5, 6]. Besides, miRNA expression patterns can be used to distinguish tumor subtypes and predict clinical outcomes [7]. Therefore, it is of great importance for the identification of GC‐associated miRNAs as biomarkers in early tumor detection, prognosis, and treatment.

The function of miR‐194 in stomach cancer still remains unclear and controversial. As early as 2012, Song *et al*. [8] have reported that the overexpression of miR‐194 predicts better prognosis in GC. After that, several studies have shown that miR‐194 inhibited GC progression via targeting FOXM1 [9], RBX1 [10], and KDM5B [11]. However, another recent study reported that miR‐194 promotes GC cell proliferation and migration by activating Wnt signaling, at least in part, via suppression of SUFU, which suggested miR‐194 plays oncogenic role in GC [12]. Many genes are negatively regulated by miR‐194, but it is not clear which target gene is the main pathway for mir‐194 to exert its anti‐tumor effect. Transcriptome sequencing is a efficient strategy that can clarify which target genes miR‐194 mainly uses to suppress tumors. Therefore, it is necessary to clarify the preferences of miR‐194 in regulating target genes.

In this study, we conducted comprehensive miRNA expression analysis in different GC subtypes using The Cancer Genome Atlas (TCGA) data and identified a panel of miRNAs (miR‐100, miR‐125b, miR‐199a, and miR‐194) that can serve as prognostic and diagnostic biomarkers for GC. In addition, RNA sequencing analysis was conducted to determine the most affected pathways and target genes by miR‐194 in GC. Our studies suggested that CCND1 can be a novel target gene of miR‐194 in GC, and miR‐194 may inhibit GC cell growth mainly through direct downregulation of CCND1 and induction of cell cycle arrest.

## Materials and methods

### Global miRNA expression analysis in TCGA

The volcano plot data showing the associations between miRNA expression and various clinical characteristics (overall survival, T stage, pathologic stage, and M stage) of GC patients from TCGA were downloaded from LinkedOmics Web tool [13]. The filter conditions were set to *P* < 0.05 and event > 300. miRNA‐seq data and the correlated detailed clinical information of GC samples were downloaded from TCGA using Sanger Box software developed by ShengXinRen. Expression level of per miRNA was calculated from log_2_ of its transcripts per million value.

### Cell culture and transfection

The human GC cell lines BGC823 and SGC7901 were purchased from the Shanghai Cell Bank of Chinese Academy of Sciences (Shanghai, China). The two GC cell lines were cultured in Dulbecco's Modified Eagle Medium containing 10% FBS, 100 U·mL^−1^ penicillin, 100 U·mL^−1^ streptomycin, and 0.03% glutamine at 37 °C in 5% CO_2_.

For siRNA transfection, the miR‐194 mimics and inhibitors were designed and synthesized by Genepharma (Shanghai, China). For miR‐194 mimics, 5′‐UGUAACAGCAAC UCCAUGUGGA‐3′, 5′‐CACAUGGAGUUGCUGUUACAUU‐3′; for miR‐194 inhibitors, 5′‐UCCACAUGGAGUUGCUGUUACA‐3′; for si‐NC, 5′‐UUCUCCGAACGUGUCA CGUTT‐3′, 5′‐ACGUGACACGUUCGGAGAATT‐3′; and for miRNA inhibitor‐NC, 5′‐CAGUACUUUUGUGUAGUACAA‐3′. Two different GC cell lines were seeded into 6‐well plates and grown overnight. The next day, when the cell plating density reached 20–30%, GC cells were transfected with siRNAs (final concentration, 50 nm) by Lipofectamine 2000 (Invitrogen, Carlsbad, CA, USA) according to the manufacturer's instructions. At 48 h post‐transfection, cells were harvested for qPCR analysis, flow cytometry assays, and RNA sequencing. At 72 h post‐transfection, cells were harvested for western blot.

### Cell proliferation assays

For cell proliferation assays, cells transfected with miRNA mimics or inhibitors for 24 h were reseeded in 96‐well plates at 2000 cells per well in a final volume of 100 μL and cultured for 4 days. The effects of miR‐194/CCND1 on cell proliferation were determined with CCK‐8 assay every 24 h. Subsequently, 10 μL of CCK‐8 solution (Biosharp, Hefei, China) was added into each well and incubated for 2 h. Optical density was measured at a wavelength of 490 nm by an automatic microplate reader (Bio‐Tek, Winooski, VT, USA). Triplicate wells were assayed for each experiment, and three independent experiments were performed. Data were expressed as the OD_490_ mean ± S.D.

### Quantitative RT–PCR

For RNA extraction, GC cells were grown in six‐well plates and transfected with siRNAs. After 48 h, the medium was removed and 800 μL TRIzol was directly added into the six‐well plate to harvest samples. Total RNA was extracted using TRIzol reagent (Invitrogen) according to the manufacturer's instructions. The isolated RNA was treated with RNase‐free DNase I (Roche, Basel, Switzerland) for 15–30 min as we described before [14]. RNA purity and concentration were checked using the NanoPhotometer spectrophotometer (Implen, Westlake Village, CA, USA). PCR was performed to ensure removal of genomic DNA by using RNA samples as the template. Reverse transcription was performed to obtain cDNA using 1 μg total RNA as the template according to the manufacturer's instructions of the PrimeScript™ RT Reagent Kit (Perfect Real Time; Takara, Dalian, China).

For quantitative RT–PCR, all the cDNA samples were diluted five times as templates of qPCR. The qPCR protocol was evaluated using one‐step TB Green PrimeScript™ RT–PCR Kit II (Takara) according to the manufacturer's instructions. The qPCR analysis was conducted on Bio‐Rad (Hercules, CA, USA) CFX Manager 3.1 Real‐Time PCR system. The cycling conditions were as follows: 9  °C for 30 s, 95 °C for 5 s, and 60 °C for 30 s. The reaction was performed for 40 cycles. The mRNA expression level was determined by using the specific primers (CCND1‐F: 5′‐TGAACTACCTGGACCGCTTC‐3′, CCND1‐R: 5′‐CCACTTGAGCTTGTTCACCA‐3′; ACTIN‐F: 5′‐ATCGTCCACCGCAAATGCTTCTA‐3′, ACTIN‐R: 5′‐AGCCATGCC AATCTCATCT TGTT‐3′).

### Western blot

At 72 h after transfecting with miRNA, GC cells were lysed in RIPA buffer added 1 mm PMSF. Approximately 100 μg of total protein was electrophoresed through 10% SDS/PAGE and was then transferred to a poly(vinylidene difluoride) (PVDF) membrane (Millipore, Boston, MA, USA). After blocking with 5% skimmed milk at 4 °C for 1 h, the membrane was incubated with CCND1 antibody (1 : 1000; Proteintech, Wuhan, China) and GAPDH (1:1000; Proteintech) at 4 °C overnight. The PVDF membrane was then washed and incubated with horseradish peroxidase (HRP)‐conjugated secondary antibody (1 : 10 000; Earthox San Francisco, CA, USA) for 1.5 h at room temperature. Detection was performed by using a SuperLumia ECL HRP Substrate Kit (Abbkine, Wuhan, China). The defined sections of the film were scanned for image capture and quantified using adobe photoshop software (Adobe Systems Incorporated, San Jose, CA, USA) and imagej software (Bio‐Rad).

### Luciferase reporter assay

For wild‐type luciferase reporter vector construction, the 3′ UTR of CCND1 (from 1900 to 2635 nt) was amplified by PCR and ligated into the pMir‐GLO luciferase reporter vector (Promega, Madison, WI, USA). For mutant luciferase reporter vector construction, the mutant CCND1 3′UTR (replaced TGTTACA with AAAAACA) was cloned into the MCS region of pMir‐GLO luciferase reporter vector with *Sac* I and *Xba* I. The primers for wild‐type and mutant CCND1 3′UTR were as follows: F: 5′‐gggagctcCTGTCCCACTC CTACGATAC‐3′, R1 (wild‐type): 5′‐tctctagaTGTAACATCAAAGGCAGAAGG‐3′, and R2 (mutant): 5′‐tctctagaTGTTTTTTCAAAGGCAGAAGGTTTGTGT‐3′. The luciferase reporter assay was conducted as we previously described before [15]. Briefly, the BGC823 cells were seeded into 12‐well tissue plates 24 h before transfection, and then co‐transfected with 5 nm siRNA and 1 mg plasmid using the Lipofectamine 2000 Reagent (Invitrogen), according to the manufacturer's instructions. After another 48 h, cells were assayed using the Dual‐Luciferase Reporter Assay System Kit (GeneCopoeia, Rockville, MD, USA). All experiments were performed in triplicate, and data were pooled from three independent experiments.

### Flow cytometry assays

After 48 h transfected with miR‐194 mimics and corresponding negative control siRNAs, SGC7901 and BGC823 cells were collected and stained with propidium iodide (BB‐4104; Best Bio, Shanghai, China) and performed in accordance with the manufacturer's protocol. Cell cycle analysis was performed on the CytoFLEX machine (Beckman, Boulevard Brea, CA, USA). The cell cycle distribution was quantified using the cytexpert software (Boulevard Brea, CA, USA).

### RNA sequencing

After 48 h transfected with miRNA mimics and corresponding negative control siRNAs, SGC7901 and BGC823 cell total RNA was extracted to perform RNA sequencing. A total amount of 1.5 µg RNA per sample was used as input material for the RNA sample preparations. The whole step of library construction and sequencing was performed at Shanghai Lifegenes Technology Co., Ltd (Shanghai, China). Random hexamer primer cDNA libraries were sequenced on Illumina HiSeq 4000 sequencing platform (Illumina, San Diego, CA, USA) according to the manufacturer's instructions for paired‐end 150 bp reads (Lifegenes, Shanghai). The RNA‐seq data were uploaded on the gene expression omnibus (GEO) section of NCBI database with Accession Number GSE134308.

### Statistical analysis

Data from at least three independent experiments performed in triplicate are presented as the mean ± SD. Comparisons were performed using Student's paired *t*‐test and Spearman's correlation test; *P* < 0.05 was considered statistically significant.

## Results

### Identification of the miRNAs associated with clinical outcome of gastric cancer

**Fig. 1:**
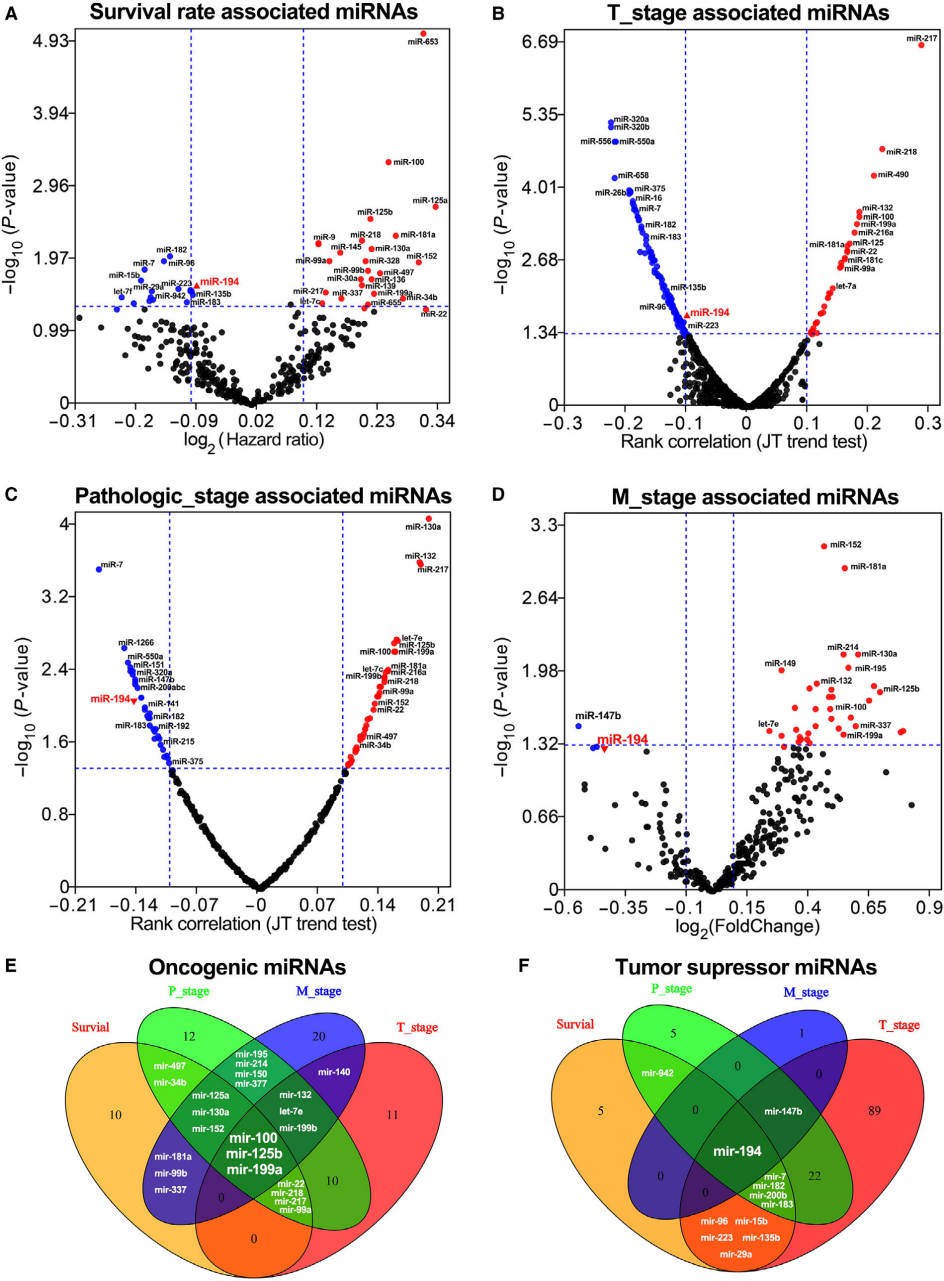
The miRNAs possessing significant associations with various clinical characteristics GC patients from TCGA were identified by miRNA expression analysis. (A) The volcano plot displays miRNAs that significantly associated with survival rate in the GC cohorts from TCGA. (B) The volcano plot displays miRNAs that significantly associated with T stage of the GC cohorts from TCGA. (C) The volcano plot displays miRNAs that significantly associated with pathological stage of the GC cohorts from TCGA. (D) The volcano plot displays miRNAs that significantly associated with M stage of the GC cohorts from TCGA. (E) The Venn diagram displays miRNAs that predict poor prognosis in GC. (F) The Venn diagram displays miRNAs that predict favorable prognosis in GC (*P* < 0.05).

The association between miRNA expression pattern and clinical outcomes was analyzed using LinkedOmics database, which is Web‐based tools to deliver fast and customizable functionalities based on TCGA data [13]. The significances (−log_10_ *P* value) of the correlation between the expression of all miRNAs and the overall survival, T stage (or pathological stage), and M stage of GC were shown in the four volcano plots of Fig. 1. The results showed that GC patients with higher expression of miR‐100, miR‐653, miR‐125a, etc., or lower expression of miR‐182, miR‐7, miR‐96, etc., were associated with a shorter overall survival time (*P* < 0.05, Fig. 1A). And the expression of miR‐217, miR‐181a, miR‐132, etc., was positively correlated with the T stage of GC, while the expression of miR‐320a, miR‐182, miR‐194, etc., was negatively correlated with the T stage of GC (*P* < 0.05, Fig. 1B). The expression of miR‐130a, miR‐217, miR‐132, etc., was positively related to the pathological stage of GC, while the expression of miR‐320a, miR‐182, miR‐194, etc., was negatively related to the pathological stage of GC (*P* < 0.05, Fig. 1C). In addition, we also note that GC tissues in M1 stage usually possessed higher expression of miR‐152, miR‐125b, miR‐100, etc., and lower expression of miR‐194 and miR‐147b (*P* < 0.05, Fig. 1D) compared with the GC tissues in M0 stage.

To determine the most appropriate diagnostic and prognostic biomarkers for GC, the Venn diagrams were plotted based on the number of miRNAs that significantly positively associated with overall survival rate, T stage, pathological stage, and M stage. The miRNAs significantly associated with at least two clinical characteristics are listed in Table 1. As shown in Fig. 1E,F, miR‐100, miR‐125b, miR‐199a, and miR‐194 were the four most promising biomarkers that can accurately predict cancer stage and reflect an individual's cancer risk in GC. Additionally, our data also suggested that miR‐100, miR‐125b, and miR‐199a were unfavorable prognostic biomarkers in GC, while miR‐194 was favorable prognostic biomarker in GC.

**Table 1:** Identification of miRNAs that significantly associated with the clinical outcomes of the GC patients from TCGA. ‘+’ means positive correlation (*P* < 0.05); ‘−’ means negative correlation (*P* < 0.05); ‘None’ means no significance (*P* ≥ 0.05).

miRNA	Significant correlation to the GC clinical characteristics and outcome		
	Survival time (rate)	Pathological stage or T stage	M stage
Oncogenic miRNA biomarkers			
miR‐100	−	+ (both)	+
miR‐125b	−	+ (both)	+
miR‐199a	−	+ (both)	+
miR‐125a	−	+ (both)	+
miR‐130a	−	+ (P stage)	+
miR‐152	−	+ (P stage)	+
miR‐132	None	+ (both)	+
Let‐7e	None	+ (both)	+
miR‐199b	None	+ (both)	+
miR‐22	−	+ (both)	None
miR‐218	−	+ (both)	None
miR‐217	−	+ (both)	None
miR‐99a	−	+ (both)	None
miR‐497	−	+ (P stage)	None
miR‐34b	−	+ (P stage)	None
miR‐181a	−	None	+
miR‐99b	−	None	+
miR‐337	−	None	+
miR‐140	None	+ (T stage)	+
miR‐195	None	+ (P stage)	+
miR‐214	None	+ (P stage)	+
miR‐150	None	+ (P stage)	+
miR‐377	None	+ (P stage)	+
Tumor suppressor miRNA biomarkers			
miR‐194	+	− (both)	−
miR‐147b	None	− (both)	−
miR‐7	+	− (both)	None
miR‐182	+	− (both)	None
miR‐200b	+	− (both)	None
miR‐183	+	− (both)	None
miR‐96	+	− (T stage)	None
miR‐15b	+	− (T stage)	None
miR‐223	+	− (T stage)	None
miR‐135b	+	− (T stage)	None
miR‐29a	+	− (T stage)	None

### Lower expression of miR‐194 predicts poorer prognosis in TCGA gastric cancer cohort

**Fig. 2:**
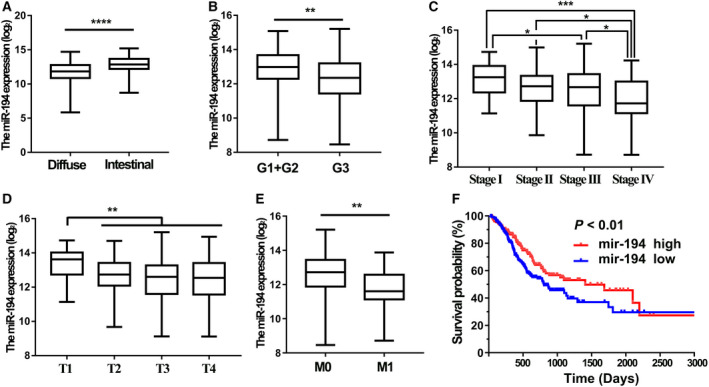
The correlations between miR‐194 expression level and clinicopathological features of the GC patients from TCGA. (A) Difference in expression levels of miR‐194 between intestinal and diffuse GC tissues; (B–E) the expression level of miR‐194 in different differentiation grade; (B) pathological stages (C), T stages (D), and M stages (E) of GC tissues from TCGA (*n* = 373); and (F) the Kaplan–Meier analysis of the overall survival time differences between GC patients with relative higher miR‐194 expression and GC patients with relative lower miR‐194 expression. The *P* values were estimated using the Mann–Whitney nonparametric test (*P < 0.05, **P < 0.01, ***P < 0.001).

In order to better understand the correlation between miR‐194 expression and clinical pathology of GC, the RNA‐seq data of miR‐194 in the stomach cancer tissues from TCGA database (*n* > 375) were further analyzed. The results showed that the expression level of miR‐194 in the diffuse type GC was significantly lower than that in the intestinal‐type GC (Fig. 2A *P* < 0.0001). Moreover, miR‐194 expression tends to be higher in moderately or highly differentiated GC tissues than that in poorly differentiated GC tissues (Fig. 2B *P* < 0.01).

Furthermore, the result showed there was a significant positive correlation between miR‐194 expression and the extent of GC progression (Fig. 2C). Similarly, miR‐194 was much higher expressed in the gastric mucosa (T1) than tumors extending beyond the gastric mucosa layer (T2 + T3 + T4; Fig. 2D). Patients with lower expression of miR‐194 were more likely to have distant tumor metastasis (Fig. 2E). More importantly, patients with lower miR‐194 expression tended to have a shorter overall survival time than those patients with higher miR‐194 expression (Fig. 2F). Collectively, these results together suggested that miR‐194 may be tumor suppressor in GC and miR‐194 could serve as an independent diagnostic and prognostic biomarker for GC.

### The preferences of miR‐194 in regulating target genes

**Fig. 3:**
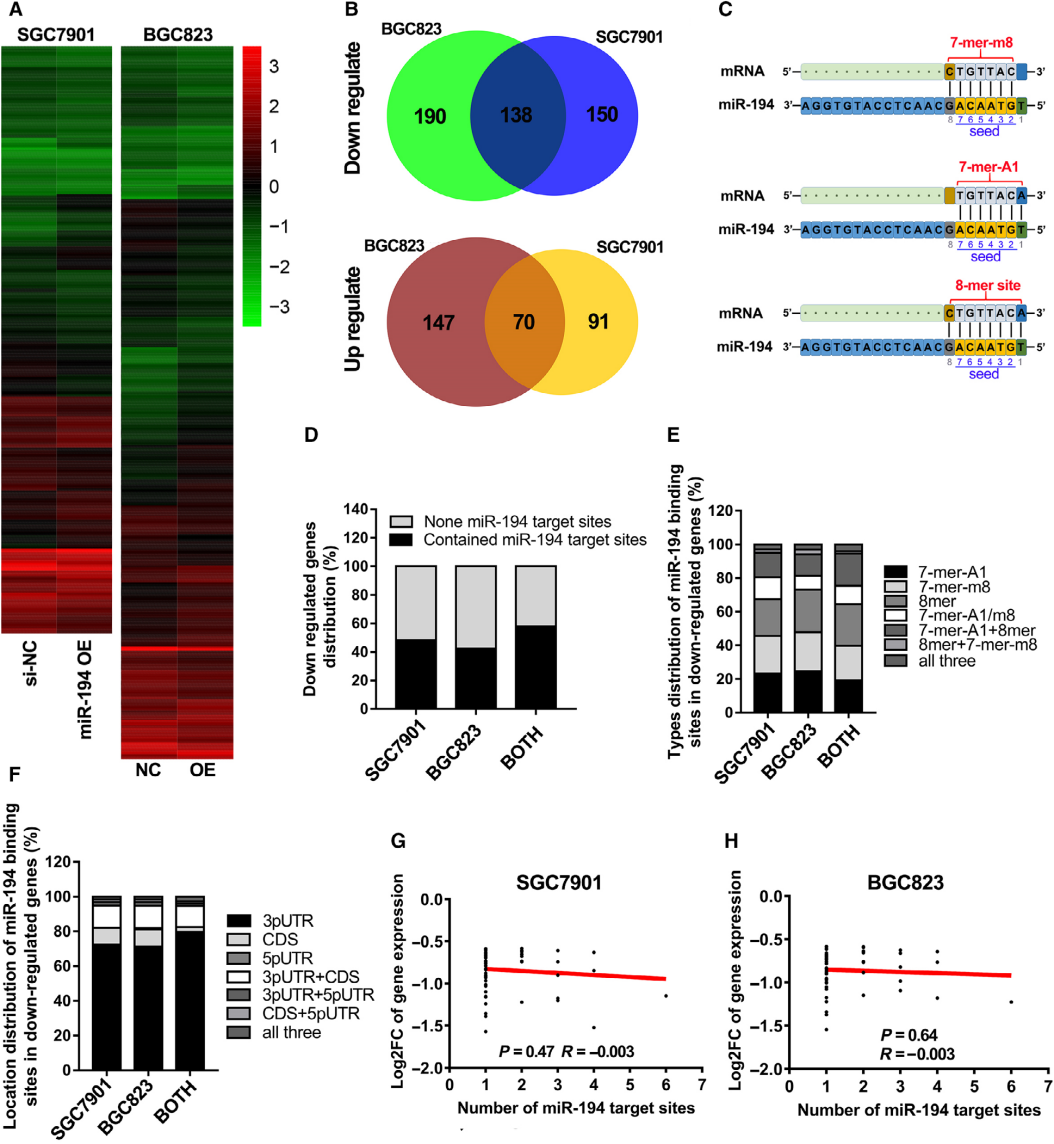
The preferences of miR‐194 in regulating target genes in GC. (A) The heat map of coding genes that regulated by miR‐194 in SGC7901 and BGC823 (FC > 1.5); (B) the coding genes that downregulated or upregulated by miR‐194 in both SGC7901 and BGC823 cells; (C) the binding mode between coding gene transcripts and miR‐194 seed region; (D) the percentage of gens that contain none miR‐194 binding site among those genes that downregulated in both SGC7901 and BGC823 cells; (E) the distribution of different binding modes between miR‐194 and target transcripts; (F) the distribution of different binding regions between miR‐194 and target transcripts; (G, H): the correlation between the FC value of target gene expression and the number of miR‐194 binding sites in target genes by analysis the RNA‐seq data of SGC7901 (G) and BGC823 (H).

To figure out the preference of miR‐194 in regulating target genes, RNA sequencing studies were performed in the two GC cell lines (SGC7901 and BGC823) that transfected with miR‐194 mimics and corresponding negative control siRNAs. The RNA sequencing data were deposited in the GEO database of NCBI GeneBank with Accession Number GSE134308. And the RNA sequencing data on identification of target genes are listed in Table S1. The heat map showed that there were hundreds of coding genes (noncoding genes were not included in this study) altering in their expression levels after treatment with miR‐194 mimics (Fig. 3A, |FC| > 1.5). Of these, ~ 138 coding genes were relatively strongly downregulated by miR‐194 mimics, while almost 70 coding genes that relatively severely upregulated by miR‐194 mimics in both SGC7901 and BGC823 cell lines (Fig. 3B). And the top 22 coding genes most strongly downregulated by miR‐194 are listed in Table 2 (log_2_ FC < −0.9).

**Table 2:** The top 23 coding genes that most downregulated by miR‐194 mimics in gastric cancer.

Gene symbols	SGC7901 (log_2_FC)	BGC823 (log_2_FC)	miR‐194 binding sites of transcripts		
			Number	Type	Position
ARL6IP5	−1.201	−1.093	3	7‐mer‐A1	3′UTR
ATP11C	−1.240	−1.076	2	7‐mer‐A1; 8‐mer	CDS (1); 3′UTR (1)
ATP6V1F	−1.160	−1.051	1	7‐mer‐A1	3′UTR
BTF3L4	−1.173	−0.980	3	7‐mer‐A1 (2); 7‐mer‐m8 (1)	3′UTR
CFL2	−1.221	−1.147	2	7‐mer‐m8	3′UTR
DUSP9	−1.358	−0.965	1	7‐mer‐m8	3′UTR
ERGIC2	−1.014	−0.831	1	8‐mer	3′UTR
FZD6	−0.901	−0.820	2	7‐mer‐A1; 8‐mer	CDS (1); 3′UTR (1)
GYG1	−0.930	−0.996	1	8‐mer	3′UTR
IL6ST	−1.521	−1.180	4	7‐mer‐A1 (3); 8‐mer (1)	3′UTR
PPP1R14B	−1.259	−1.143	1	8‐mer	CDS
GNS	−0.901	−1.291	1	8‐mer	3′UTR
RETREG1	−1.151	−0.977	2	7‐mer‐m8	CDS (1); 3′UTR (1)
SDCBP	−1.569	−1.542	1	8‐mer	3′UTR
PRKAR1A	−1.151	−1.002	1	8‐mer	3′UTR
GOT2	−0.926	−1.044	1	8‐mer	3′UTR
SLC7A5	−1.029	−1.339	1	8‐mer	3′UTR
ELOVL3	−1.387	−1.371	1	7‐mer‐m8	CDS
SRGN	−1.356	−1.174	1	8‐mer	3′UTR
TCEAL4	−0.913	−1.080	1	8‐mer	3′UTR
TEX30	−1.019	−1.033	2	7‐mer‐A1 (3); 8‐mer (1)	3′UTR
TMED5	−1.146	−1.224	6	7‐mer‐A1 (3); 7‐mer‐m8 (2);8‐mer (1)	3′UTR
YWHAZ	−1.098	−0.951	1	7‐mer‐m8	3′UTR

According to the binding sequence differences in the miR‐194 seed region, the miR‐194 binding sites in mRNAs can be divided into three types, including 7‐mer‐A1, 7‐mer‐m8, and 8‐mer (Fig. 3C). Then, the information of miR‐194 binding sites in the coding genes that were downregulated by miR‐194 was annotated using miRcode Web‐based tools. The results showed that nearly 60% of the coding genes contained miR‐194 binding sites (Fig. 3D). And the distribution proportion of the three binding modes is very close, suggested that any type of the three binding manners between miR‐194 and mRNAs would be effective (Fig. 3E). Next, the binding location preference of miR‐194 in mRNA sequence was further analyzed. The results showed that almost 86% of miR‐194 binding sites were located at 3′ UTR of target genes (Fig. 3F). In addition, no significant correlation was observed between expression alteration (log_2_FC) and the number of miR‐194 binding sites in both SGC7901 and BGC823 cell lines (Fig. 3G,H).

### miR‐194 negatively regulated CCND1 expression by binding on the 3′ UTR in GC

**Fig. 4:**
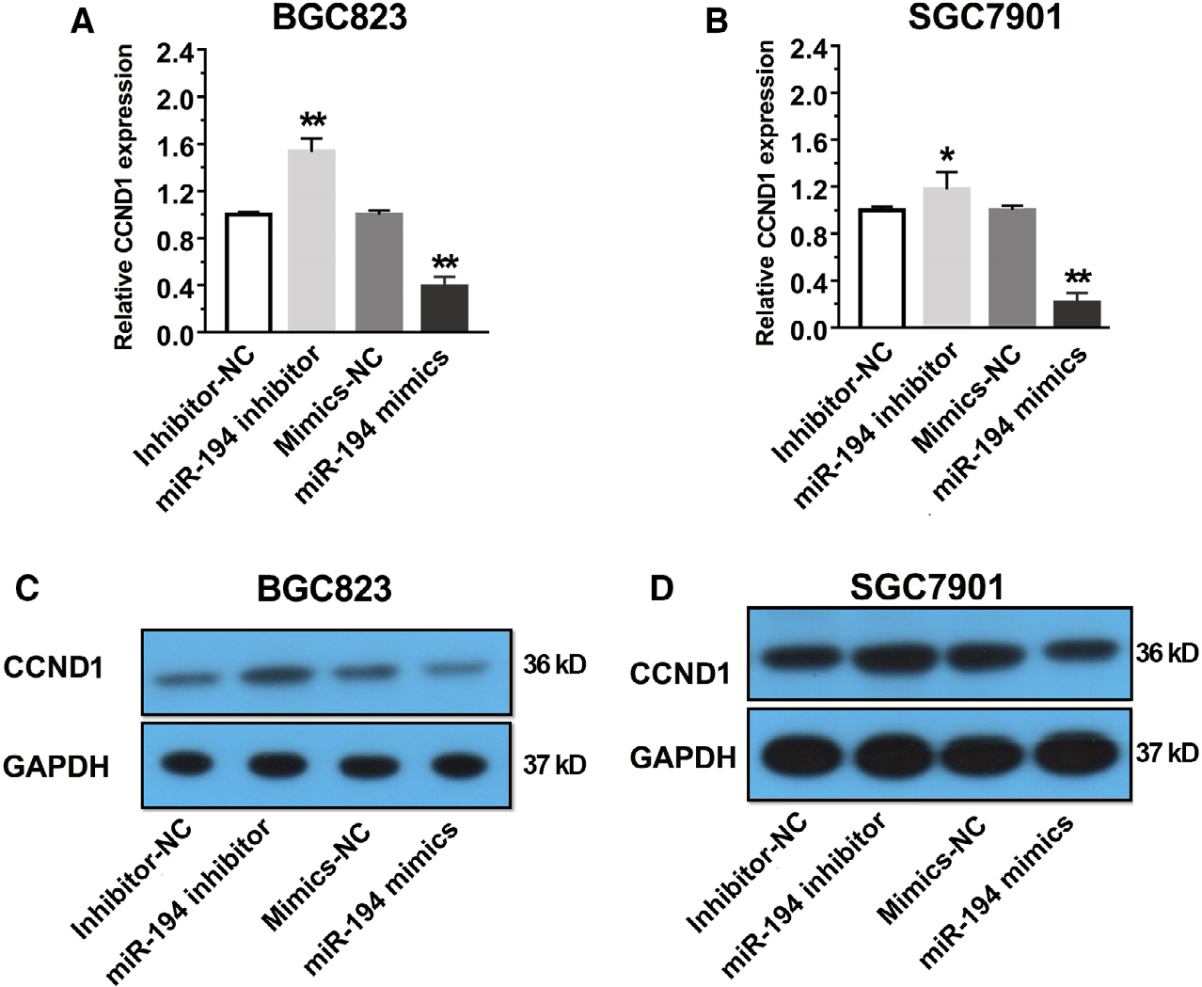
CCND1 was negatively regulated by miR‐194 in GC. (A) Determination of CCND1 expression level in the BGC823 cells that transfected with miR‐194 mimics and inhibitors by qRT–PCR assay. (B) Determination of CCND1 expression level in the SGC7901 cells that transfected with miR‐194 mimics and inhibitors by qRT–PCR assay. (C) Determination of CCND1 protein level in the BGC823 cells that transfected with miR‐194 mimics and inhibitors by western blot assay. (D) Determination of CCND1 protein level in the BGC823 cells that transfected with miR‐194 mimics and inhibitors by western blot assay. Results are expressed as means ± SD and are representative of three independent experiments. Data were analyzed by one‐way ANOVA method (*P < 0.05, **P < 0.01).

KEGG pathway analysis was conducted to determine the most affected pathways by miR‐194 in GC. As shown in Fig. S1, the cell cycle pathway was one of the most affected pathways by miR‐194. RNA‐seq data showed that CCND1 was strongly downregulated in both SGC7901 and BGC823 cell lines (log_2_FC value was −0.84 in SGC7901, −0.71 in BGC823). To confirm the RNA‐seq results, the CCND1 expression in two GC cell lines after treatment with miR‐194 mimics and inhibitors was investigated via qPCR analysis and western blot assay. The results showed that the overexpression of miR‐194 significantly decreased CCND1 expression in GC, and miR‐194 inhibitor significantly increased CCND1 expression in GC (Fig. 4). These results strongly indicated that CCND1 was negatively regulated by miR‐194.

**Fig. 5:**
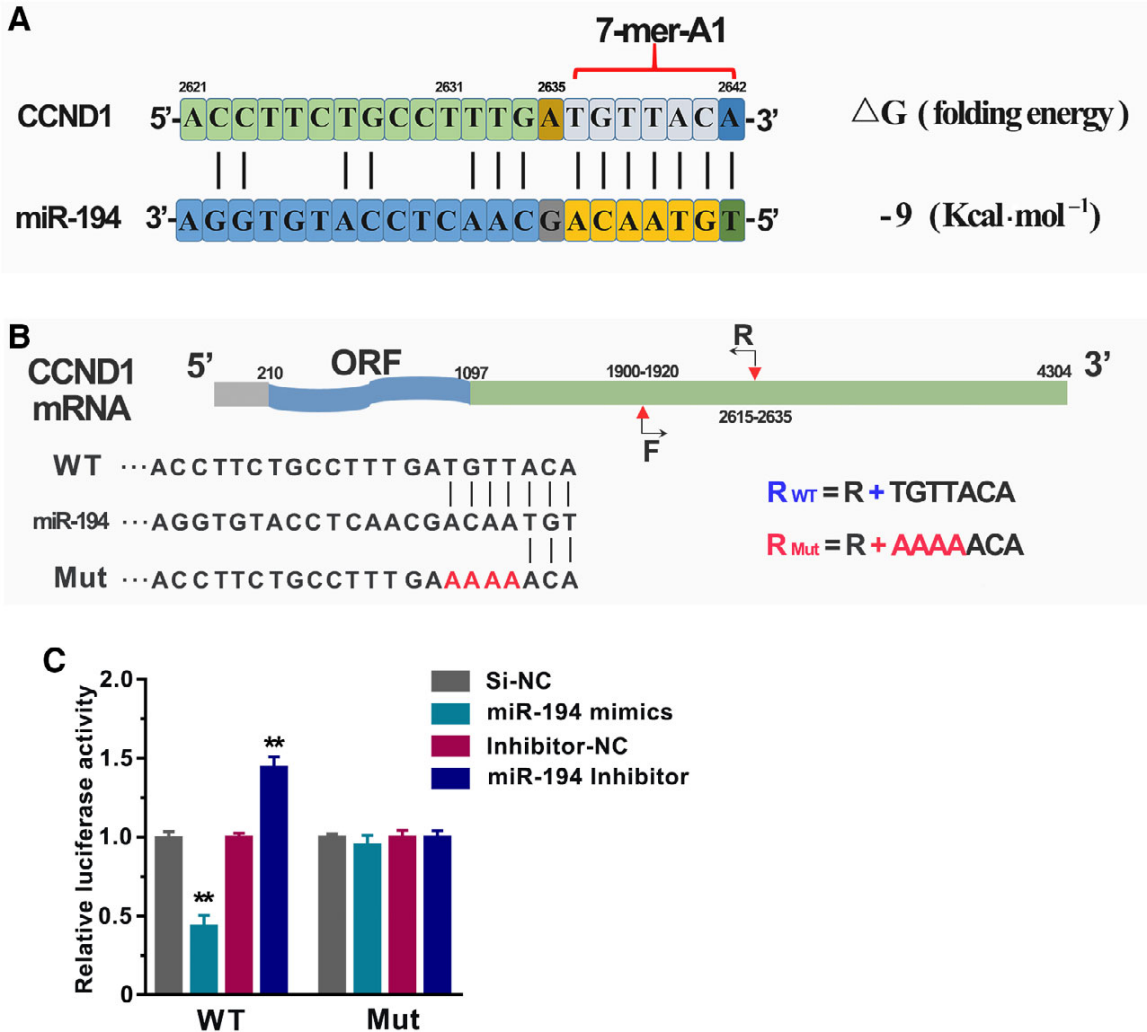
The CCND1 gene was a target gene of miR‐194 in GC. (A) The binding mode and folding energy between miR‐194 and CCND1 were predicted by MiRcode and RNA22 Web‐based tool. (B) The schematic diagram of construction of wild‐type luciferase reporter vector and the mutant luciferase reporter vector. (C) The BGC823 cells were co‐transfected with miR‐194 mimics (or inhibitors) and wild‐type (or mutant) luciferase reporter vector. After 48 h of incubation, luciferase activity was measured. Results are expressed as means ± SD and are representative of three independent experiments. Data were analyzed by one‐way ANOVA method (**P < 0.01).

Based on the above results, the information of miR‐194 binding site in the CCND1 transcript was further analyzed using the miRcode Web‐based tools and the folding energy between miR‐194 and CCND1 transcript was predicted using RNA22 Web‐based tools [16, 17]. As shown in Fig. 5A, there was only one miR‐194 binding site (7‐mer‐A1) in the 3′ UTR of CCND1. The folding energy between miR‐194 and CCND1 transcript was −9 Kcal·mol^−1^, suggesting that the negative regulation of miR‐194 on CCND1 expression might be through regulating mRNA stability by the direct binding on CCND1 transcripts. To further validate this hypothesis, luciferase reporter assay was performed. Since the length of 3′ UTR of CCND1 was too large to be amplified, the 735‐length sequence fragment (from 1900 to 2642 nt) was selected to clone into the luciferase reporter vector with original sequence (wild‐type) or with the 4 nt changed (mutant) in the miR‐194 binding site (Fig. 5B). As expected, comparing to the BGC823 cells that transfected with negative control siRNA and wild‐type luciferase vector, the significant decrease and increase were observed in the BGC823 cells that transfected with miR‐194 mimics and inhibitors, respectively. However, no obvious changes were detected in the BGC823 cells that transfected with mutant CCND1 3′UTR luciferase vector (Fig. 5C). These results strongly suggested that miR‐194 negatively regulates CCND1 expression at post‐transcriptional level.

### miR‐194 suppressed GC cell proliferation through induction of GC cell cycle arrest

**Fig. 6:**
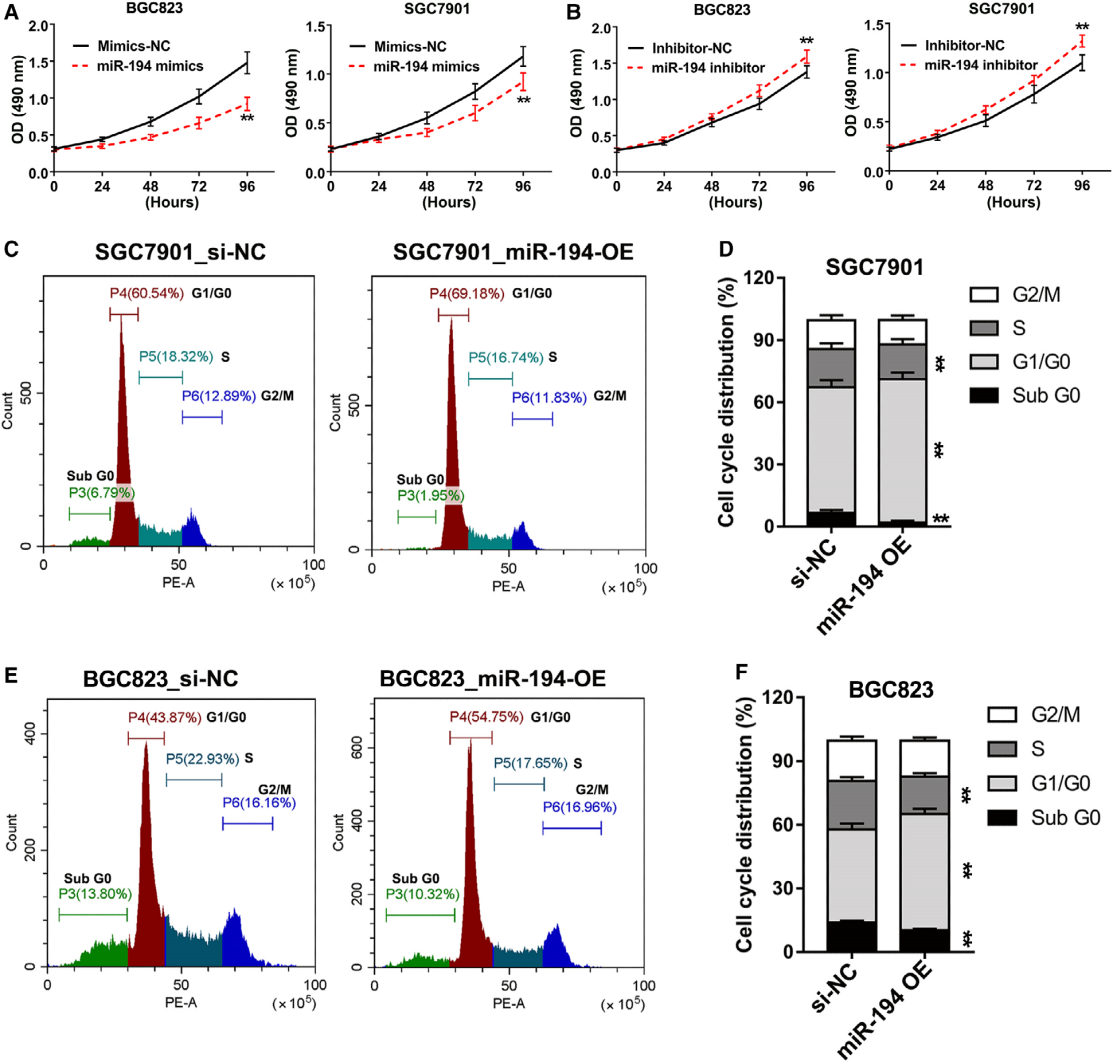
The GC cell growth and cell cycles were impaired by miR‐194. (A) The CCK‐8 proliferation assay in SGC7901 and BGC823 cells that transfected with miR‐194 mimics. (B) The CCK‐8 proliferation assay in SGC7901 and BGC823 cells that transfected with miR‐194 inhibitors. (C) Flow cytometric analysis was performed in SGC7901 cells that transfected with miR‐194 mimics. (D) The data of cell cycle analysis were described as percentage distribution of cells in sub‐G0, G0/G1, S, and G2/M phases of the cell cycle. (E) Flow cytometric analysis was performed in SGC7901 cells that transfected with miR‐194 mimics. (F) The data of cell cycle analysis were described as percentage distribution of cells in sub‐G0, G0/G1, S, and G2/M phases of the cell cycle. All flow cytometry assays were reproducible in three independent experiments. Results are expressed as means ± SD and are representative of three independent experiments. Data were analyzed by one‐way ANOVA method (**P < 0.01).

The cyclin protein CCND1 plays an essential role in the cell cycle process. Increasing studies have demonstrated that downregulation of CCND1 induces G1 phase arrest and then impairs cell growth [18]. Therefore, we performed the cell proliferation assay in SGC7901 and BGC823 cells that transfected with miR‐194 mimics and inhibitors. The results showed that transfection of miR‐194 mimics markedly suppressed the cell proliferation in GC, while transfection of miR‐194 inhibitors slightly promoted the cell proliferation in GC (Fig. 6A,B). On the other hand, the flow cytometry assays were conducted in the two GC cell lines after treatment with miR‐194 mimics and corresponding negative control siRNAs, of which the results showed that compared to NC group, miR‐194 significantly decreased the number of the sub‐G0 phase and S phase cells, but increased the number of G1 phase cells in both SGC7901 and BGC823 (Fig. 6C–F). These results together suggested that miR‐194 inhibits GC cell growth via induction of cell cycle arrest.

## Discussion

Gastric cancer, a leading cause of cancer‐related deaths, is a heterogeneous disease with many kinds of subtypes. Mounting evidences show that different subtype of GCs usually possess distinct clinical outcomes [19, 20]. In the past, only the coding genes were developed as biomarkers for tumor diagnosis. However, nowadays increasing noncoding RNAs are found to be functional in multiple biological process. And more and more noncoding genes have been developed as biomarkers for tumor diagnosis. As a member of noncoding RNAs, miRNAs usually have specific expression profiles in cancer cells and tissues, and can enter the body fluid circulation. Therefore, the development of miRNAs as diagnostic markers is gaining more and more attention.

To develop biomarkers that can accurately predict stomach cancer stage and reflect an individual's stomach cancer risk, miRNA expression analysis was performed using the available TCGA data. And our global miRNA expression analysis (RNA‐seq data of 391 GC patients) identifies miR‐194, miR‐100, miR‐125b, and miR‐199a as the four most promising biomarkers for GC (Fig. 1). It is worth noting that three of them (miR‐100, miR‐125b, and miR‐199a) have also been identified by another independent research using miRNA microarray data of 353 GC samples (from 182 GC patients), which suggested our global miRNA expression analysis results are convincing [21].

Although the role of miR‐194 in GC remains controversial, our findings strongly demonstrated that miR‐194 plays a tumor suppressor role in GC. The expression pattern and function of miR‐100, miR‐125b, and miR‐199a in GC have widely been studied. Wu *et al*. [22] have reported that miR‐125b promotes GC progression by targeting PPP1CA‐Rb signal pathways and predicts a poor prognosis in GC. Another study by Sui *et al*. reported that miR‐125b is associated with poor prognosis and trastuzumab resistance in HER2‐positive GC. Chang *et al*. demonstrated that miR‐125b promotes invasion and metastasis of GC by targeting STARD13 and NEU1. Yang *et al*. [23, 24] proved that upregulation of miR‐100 in GC plays critical roles in primary human gastric tumorigenesis and progression. Similarly, miR‐199b has also been reported to play oncogenic roles in GC [25, 26, 27].

As mentioned in the Introduction section, miRNAs usually have preferences in the regulation of their target genes. Therefore, our next tasks are to figure out the preference of miR‐194 in regulating target genes and to elucidate the molecular mechanisms by which miR‐194 inhibits GC. RNA sequencing studies in two GC cell lines showed that miR‐194 tends to regulate target genes by binding on the 3′UTR in a 7‐mer‐A1 or 7‐mer‐m8 or 8‐mer manner. Nearly 138 coding genes are strongly downregulated by miR‐194 in both SGC7901 and BGC823 GC cell lines, including CCND1. Previous studies have shown that miR‐194 inhibited tumor progression by downregulating FOXM1 [9], RBX1 [10], and KDM5B [11]. Interestingly, based on analysis of the miR‐194 binding sites in those genes, it is notable that all the genes except FOXM1 contained miR‐194 binding sites (7‐mer‐A1: KDM5B; 7‐mer‐m8: RBX1, KDM5B). Correspondingly, RBX1 and KDM5B were indeed slightly downregulated, while almost has no alteration in FOXM1 expression after treatment with miR‐194 mimics (data not shown). Therefore, miR‐194‐RBX1 and miR‐194‐KDM5B axis might not be at least the primary pathways mediated by miR‐194 in GC inhibition.

To our knowledge, our study provided the first comprehensive transcriptome analysis about miR‐194 in GC. The RNA‐seq analysis showed that CCND1 was one of the most downregulated genes by miR‐194 in GC. The KEGG pathway analysis showed that cell cycle was one of the most affected pathways by miR‐194 in GC. CCND1 was proved to be a novel target gene of miR‐194 in GC. Therefore, our finding suggested that miR‐194 inhibits the progression of GC mainly through affecting the cell cycle pathway.

In addition, the quantitative real‐time PCR (qRT–PCR) assay, western blot assay, and luciferase reporter assay together suggested that miR‐194 negatively regulated CCND1 expression by binding on its 3′UTR region. Mounting evidences showed that the downregulation of CCND1 by miRNAs, such as miR‐155 [18], miR‐490‐5p [28], and miR‐193b [29], would simultaneously induce cell cycle arrest at G1 phase. Consistent with previous reports, our findings also showed that miR‐194 inhibits GC cell growth and induces cell cycle arrest at G1 phase. Previous published studies reported that lower expression of miR‐194 tended to have larger tumor size [8]. It is well known that cell cycle process was closely related to cancer cell growth. Given this, our results may at least partially provide a possible explanation about this phenomenon.

In summary, our study provides a comprehensive miRNA expression analysis in GC using the TCGA data. miR‐100, miR‐125b, miR‐199a, and miR‐194 are identified to be the 4 most promising miRNAs that can serve as prognostic and diagnostic biomarkers for GC. Additionally, our findings would at least partially clarify this controversy regarding the role of miR‐194 in GC. miR‐194 suppresses GC cell growth mainly through targeting CCND1 and induction of cell cycle arrest.

## Conflict of Interest

The authors declare no conflict of interest.

## Author contributions

DL conceived and designed the study. DL wrote the paper. JW performed most of the experiments. SQ, MZ, XH, JS and RS carried out initial data analyses and performed partial of the experiments. All authors contributed to drafting the manuscript. All authors have read and approved the final submitted manuscript.

## Supporting information


**Fig. S1.** The KEGG pathways that affected by miR‐194 mimics were analyzed using the RNA‐seq data in GSE134308.

**Table S1.** The RNA‐seq data of exogenous overexpression of miR‐194‐5p in SGC7901 and BGC823 cell lines.

## Data Availability

The datasets generated during the current study are available in the GEO repository, and the Accession Number is GSE134308.

## References

[1] Shah S , Underwood FE , Ng WK , Chan WY , Castaneda D , Riyat N , Azhari H , Ng SC and Kaplan G (2018) The Global incidence of gastric cancer in the 21st century: a systematic review of population‐based studies from 2007–2017. Gastroenterology 154, S986.

[2] Sano T (2017) Gastric cancer: Asia and the world. Gastric Cancer 20, S1–S2. 10.1007/s10120-017-0694-9 28144923

[3] Yusefi A , Bagheri Lankarani K , Bastani P , Radinmanesh M and Kavosi Z (2018) Risk factors for gastric cancer: a systematic review. Asian Pac J Cancer Prev 19, 591–603. 29579788 10.22034/APJCP.2018.19.3.591 PMC5980829

[4] Ling H , Fabbri M and Calin GA (2013) MicroRNAs and other non‐coding RNAs as targets for anticancer drug development. Nat Rev Drug Discov 12, 847. 24172333 10.1038/nrd4140 PMC4548803

[5] Reddy KB (2015) MicroRNA (miRNA) in cancer. Cancer Cell Int 15, 38. 25960691 10.1186/s12935-015-0185-1 PMC4424445

[6] Li D , Wang J , Zhang M , Hu X , She J , Qiu X , Zhang X , Xu L , Liu Y and Qin S (2020) LncRNA MAGI2‐AS3 is regulated by BRD4 and promotes gastric cancer progression via maintaining ZEB1 overexpression by sponging miR‐141/200a. Mol Ther Nucleic Acids 19, 109–123. 31837602 10.1016/j.omtn.2019.11.003 PMC6920306

[7] Ferracin M , Laprovitera N , Porcellini E and Grzes M (2018) Cancer site‐specific multiple microRNA quantification by droplet digital PCR. Front Oncol 8, 447. 30374423 10.3389/fonc.2018.00447 PMC6196277

[8] Song Y , Zhao F , Wang Z , Liu Z , Chiang Y , Xu Y , Gao P and Xu H (2012) Inverse association between miR‐194 expression and tumor invasion in gastric cancer. Ann Surg Oncol 19, S509–S517. 21845495 10.1245/s10434-011-1999-2

[9] Li Z , Ying X , Chen H , Ye P , Shen Y , Pan W and Zhang L (2014) MicroRNA‐194 inhibits the epithelial‐mesenchymal transition in gastric cancer cells by targeting FoxM1. Dig Dis Sci 59, 2145–2152. 24748184 10.1007/s10620-014-3159-6

[10] Chen X , Wang Y , Zang W , Du Y , Li M and Zhao G (2015) miR‐194 targets RBX1 gene to modulate proliferation and migration of gastric cancer cells. Tumor Biol 36, 2393–2401. 10.1007/s13277-014-2849-1 25412959

[11] Bao J , Zou JH , Li CY and Zheng GQ (2016) miR‐194 inhibits gastric cancer cell proliferation and tumorigenesis by targeting KDM5B. Eur Rev Med Pharmacol Sci 20, 4487–4493. 27874950

[12] Peng Y , Zhang X , Ma Q , Yan R , Qin Y , Zhao Y , Cheng Y , Yang M , Wang Q and Feng X (2017) MiRNA‐194 activates the Wnt/β‐catenin signaling pathway in gastric cancer by targeting the negative Wnt regulator SUFU, Cancer Lett 385, 117–127. 27810403 10.1016/j.canlet.2016.10.035

[13] Vasaikar SV , Straub P , Wang J and Zhang B (2018) LinkedOmics: analyzing multi‐omics data within and across 32 cancer types. Nucleic Acids Res 46, D956–D963. 29136207 10.1093/nar/gkx1090 PMC5753188

[14] Qin SS , Tang YH , Chen YP , Wu PZ , Li MR , Wu GJ and Jiang HW (2016) Overexpression of the starch phosphorylase‐like gene (PHO3) in lotus japonicus has a profound effect on the growth of plants and reduction of transitory starch accumulation. Front Plant Sci 7, 1315. 27630651 10.3389/fpls.2016.01315 PMC5005325

[15] Li D , Cheng P , Wang J , Qiu X , Zhang X , Xu L , Liu Y and Qin S (2019) IRF6 is directly regulated by ZEB1 and ELF3, and predicts a favorable prognosis in gastric cancer. Front Oncol 9, 220. 31019894 10.3389/fonc.2019.00220 PMC6458252

[16] Jeggari A , Marks DS and Larsson E (2012) miRcode: a map of putative microRNA target sites in the long non‐coding transcriptome. Bioinformatics 28, 2062–2063. 22718787 10.1093/bioinformatics/bts344 PMC3400968

[17] Hofacker IL , Fontana W , Stadler PF , Bonhoeffer LS and Schuster P (1994) Fast folding and comparison of RNA secondary structures. Monatsh Chem 125, 167–188.

[18] Ma Z , Ma Y , Xia Q , Li Y , Li R , Chang W , Chen J , Leng Z and Tao K (2016) MicroRNA‐155 expression inversely correlates with pathologic stage of gastric cancer and it inhibits gastric cancer cell growth by targeting cyclin D1. J Cancer Res Clin Oncol 142, 1201–1212. 26955820 10.1007/s00432-016-2139-y PMC11819369

[19] Abbas M , Faggian A , Sintali DN , Khan GJ , Naeem S , Shi M and Chen D (2018) Current and future biomarkers in gastric cancer. Biomed Pharmacother 103, 1688–1700. 29864959 10.1016/j.biopha.2018.04.178

[20] Yasui W , Oue N , Aung PP , Matsumura S , Shutoh M and Nakayama H (2005) Molecular‐pathological prognostic factors of gastric cancer: a review. Gastric Cancer 8, 86–94. 15864715 10.1007/s10120-005-0320-0

[21] Ueda T , Volinia S , Okumura H , Shimizu M , Taccioli C , Rossi S , Alder H , Liu Cg , Oue N and Yasui W (2010) Relation between microRNA expression and progression and prognosis of gastric cancer: a microRNA expression analysis. Lancet Oncol 11, 136–146. 20022810 10.1016/S1470-2045(09)70343-2 PMC4299826

[22] Wu JG , Wang JJ , Jiang X , Lan JP , He XJ , Wang HJ , Ma YY , Xia YJ , Ru GQ and Ma J (2015) MiR‐125b promotes cell migration and invasion by targeting PPP1CA‐Rb signal pathways in gastric cancer, resulting in a poor prognosis. Gastric Cancer 18, 729–739. 25240408 10.1007/s10120-014-0421-8

[23] Yang G , Gong Y , Wang Q , Wang Y and Zhang X (2015) The role of miR‐100‐mediated Notch pathway in apoptosis of gastric tumor cells. Cell Signal 27, 1087–1101. 25703026 10.1016/j.cellsig.2015.02.013

[24] Yang G , Gong Y , Wang Q , Wang L and Zhang X (2017) miR‐100 antagonism triggers apoptosis by inhibiting ubiquitination‐mediated p53 degradation. Oncogene 36, 1023. 27524417 10.1038/onc.2016.270

[25] Song G , Zeng H , Li J , Xiao L , He Y , Tang Y and Li Y (2010) miR‐199a regulates the tumor suppressor mitogen‐activated protein kinase kinase kinase 11 in gastric cancer. Biol Pharm Bull 33, 1822–1827. 21048306 10.1248/bpb.33.1822

[26] He XJ , Ma YY , Yu S , Jiang XT , Lu YD , Tao L , Wang HP , Hu ZM and Tao HQ (2014) Up‐regulated miR‐199a‐5p in gastric cancer functions as an oncogene and targets klotho. BMC Cancer 14, 218. 24655788 10.1186/1471-2407-14-218 PMC3994330

[27] Wang Z , Ma X , Cai Q , Wang X , Yu B , Cai Q , Liu B , Zhu Z and Li C (2014) MiR‐199a‐3p promotes gastric cancer progression by targeting ZHX1. FEBS Lett 588, 4504–4512. 25448600 10.1016/j.febslet.2014.09.047

[28] Zhao L , Tang XP , Luo RG , Duan J , Wang YC and Yang BB (2018) MicroRNA‐490‐5P targets CCND1 to suppress cellular proliferation in glioma cells and tissue through Cell cycle arrest. Curr Neurovasc Res 15, 246–255. 30101705 10.2174/1567202615666180813130143

[29] Wang L , Zhang Y , Zhao L , Liu S , Yu S , Ma Y and Sun G (2016) MicroRNA‐193b inhibits the proliferation, migration and invasion of gastric cancer cells via targeting cyclin D1. Acta Histochem 118, 323–330. 27071318 10.1016/j.acthis.2016.02.001

