# Supplementary figures and images for: miRNA‐194 predicts favorable prognosis in gastric cancer and inhibits gastric cancer cell growth by targeting CCND1
Source: FEBS Open Bio. 2021 Mar 23;11(7):1814–26. doi: 10.1002/2211-5463.13125 (PMC8255842; doi:10.1002/2211-5463.13125)

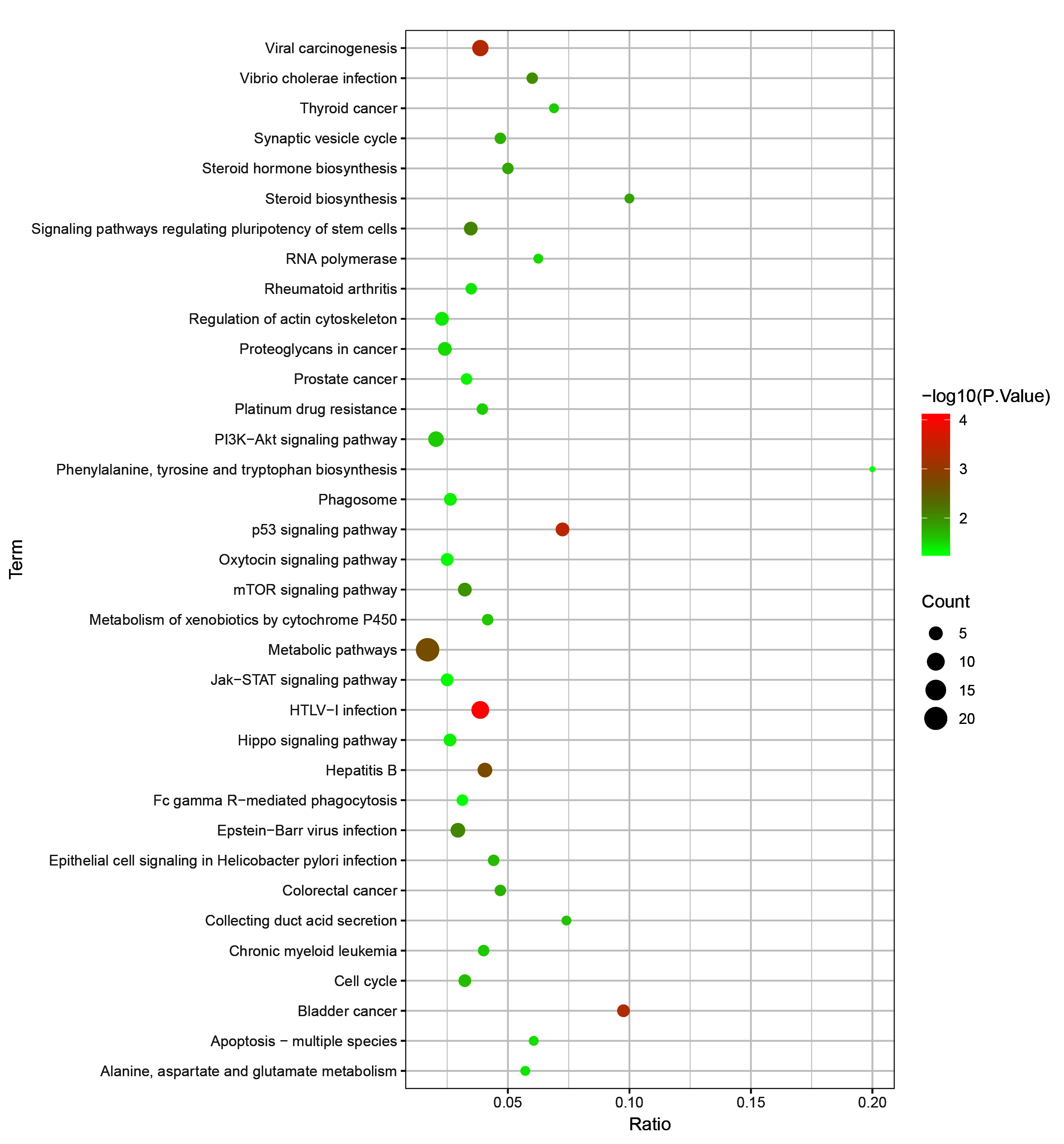

Supplement: Supplementary file 1 — Fig. S1. The KEGG pathways that affected by miR‐194 mimics were analyzed using the RNA‐seq data in GSE134308. [file FEB4-11-1814-s001.tif]
